# Effect of high-amylose *starch branching enzyme II* wheat mutants on starch digestibility in bread, product quality, postprandial satiety and glycaemic response†

**DOI:** 10.1039/d1fo03085j

**Published:** 2022-01-26

**Authors:** Marina Corrado, Jennifer H. Ahn-Jarvis, Brendan Fahy, George M. Savva, Cathrina H. Edwards, Brittany A. Hazard

**Affiliations:** Food Innovation and Health, Quadram Institute Bioscience, Norwich Research Park UK cathrina.edwards@quadram.ac.uk; Designing Future Wheat and Molecules from Nature, John Innes Centre, Norwich Research Park UK

## Abstract

High-amylose *starch branching enzyme II* (*sbeII*) mutant wheat has potential to be low-glycaemic compared to conventional wheat; however, the effects of bread made from *sbeII* wheat flour on glycaemic response and product quality require investigation. We report the impact of white bread made from *sbeII* wheat flour on *in vitro* starch digestibility and product quality, and on postprandial glycaemia *in vivo*, compared to an isoglucidic wild-type (WT) control white bread. Starch in *sbeII* bread was ∼20% less susceptible to *in vitro* amylolysis leading to ∼15% lower glycaemic response measured *in vivo*, compared to the WT control bread, without major effects on bread appearance or texture, measured instrumentally. Despite the early termination of the *in vivo* intervention study due to the COVID-19 outbreak (*n* = 8 out of 19), results from this study indicate that *sbeII* wheat produces bread with lower starch digestibility than conventional white bread.

## Abstract 2

Impact of white bread made from *sbeII* wheat flour on postprandial glycaemic response.

## Graphical abstract

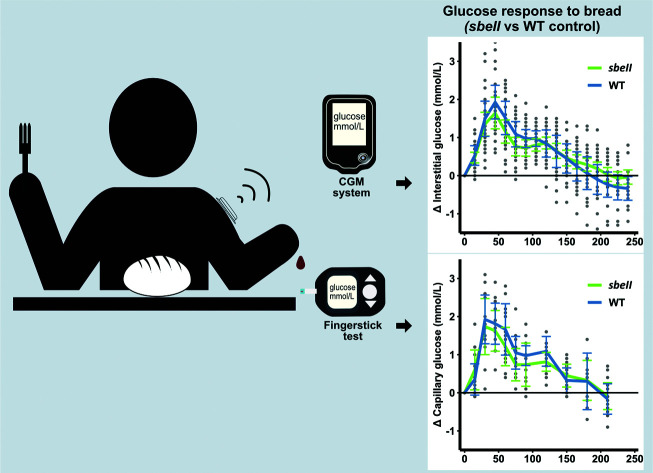

## Introduction

Large dietary intakes of high glycaemic starchy foods can exacerbate the metabolic abnormalities of insulin resistance and over time, lead to abnormal glucose tolerance, insulin sensitivity and hyperglycaemia.^1^ Approximately 65% of individuals with impaired glucose tolerance eventually develop type 2 diabetes,^2^ which affects ∼6% of the UK population.^3^ Carbohydrates (including starch) account for around 45% of dietary energy intake,^4^ however, not all carbohydrates have the same physiological effect. For instance, the postprandial rise in blood glucose and insulin concentrations varies considerably between starch-rich foods and is strongly influenced by the rate and extent of starch digestion by α-amylase in the small intestine.^5^ Foods eliciting low glycaemic responses (as measured by glycaemic index) are needed for dietary prevention and management of obesity, type 2 diabetes, and cardiovascular disease.^6,7^ The manipulation of starch structure through non-transgenic genetic approaches provides new opportunities to develop slowly digested carbohydrate staple foods with lower glycaemic potency.

Starch structure can be manipulated *in planta* using Targeting Induced Local Lesions in Genomes (TILLING) technology^8^ to induce mutations in genes involved in starch biosynthesis.^9^ Wheat carrying mutations in *starch branching enzyme II* (*sbeII*) genes have been generated using TILLING, yielding starch with a higher proportion of amylose and increased resistance to amylase digestion, compared to conventional wheat starch.^10,11^ This allows the production of high-amylose starch-based food products without the need of post-extraction processing or supplementation with starches from other botanical sources, which was shown to be a suitable approach to lower glycaemic response to high glycaemic foods, such as bread.^12,13^

Previously, a *sbeII* mutant wheat was used to produce a semolina pudding with lower starch digestibility than the control pudding made from WT wheat. When tested in humans, however, the Glycaemic Index of the *sbeII* pudding was not significantly different from the WT control.^14^ Recently, two studies have investigated glycaemic responses to pasta and bread made from *sbeII* mutant wheat. Sissons *et al.* (2020),^15^ reported that a serving of pasta (spaghetti) made from a *sbeIIa* durum wheat mutant with starch made of ∼58% amylose, corresponding to ∼7% RS, led to a lower starch susceptibility to amylase digestion *in vitro* and lower glycaemic index measured *in vivo*, compared to the WT control. Belobrajdic *et al.* (2019),^16^ reported that bread made from high-amylose wheat flour lowered the postprandial glycaemic response by 39% and the insulinemic response by 24%, however the bread provided ∼24 g less starch per 100 g and ∼7.5% less energy (kJ per 100 g) than the control bread. Therefore, it is not clear if the attenuated postprandial glycaemic response following high-amylose bread in the Belobrajdic *et al.* study was due to the lower amount of starch per serving (glycaemic load) or due to a change in the intrinsic starch digestibility.

Further studies are needed to better understand how and to what extent *sbeII* wheat can be used to attenuate the glycaemic potency of different food products. The aim of the present study was to determine the effect of *sbeII* wheat in bread on postprandial glycaemic response and satiety, compared to WT control bread. Unlike a Glycaemic Index study, this postprandial study was focussed on understanding the effects of *sbeII* starch in bread on glycaemic response curve size and shape, compared to a conventional white bread, to inform on the potential health effect of replacing conventional white bread with *sbeII* bread.

The bread rolls were prepared to deliver the same amount of starch per serving (75 g), such that any observed differences in the glycaemic responses to these bread rolls reflect differences in intrinsic starch amylolysis (measured *in vitro*). A preliminary sensory assessment (palatability) and quality analysis of *sbeII* bread was also conducted however, due to the small number of participants completing the study, results were not conclusive. The methodology was reported in ESI.†

An additional aspect of this study was the measurement of postprandial glucose using a continuous glucose monitoring system (CGM) to assess glycaemic responses, in parallel with fingerprick (FP) sampling for capillary glucose measurements. CGMs were fitted to the upper arm and used enzymatic-amperometric technology to measure glucose concentrations in interstitial fluid (IF) over several days. Recent studies have used CGMs to investigate the effect of dietary interventions on glucose responses in healthy individuals.^17,18^ Dye *et al.*^19^ and Bajka *et al.*^20^ reported a good correlation between capillary and IF glucose concentrations despite a time lag between measurements, likely due to physiological differences in the regulation of glucose exchange between the blood and the interstitial compartments. In the present study, the relationship between CGM and FP measurements was explored.

## Methods

### Bread formulation and process

A *sbeII* mutant bread wheat and a WT control bread wheat (*Triticum aestivum* L. ssp. *aestivum*) were sown in spring 2018 in a field trial using a randomised block design at the John Innes Centre Church Farm field station (Bawburgh, UK). Wheat was harvested in summer 2018, grains were transported to Campden BRI (Chipping Campden, UK). Following tempering to 16.4% humidity, grains were debranned and milled on a Bühler mill with feeding rate of 100 g min^−1^, then sieved through 140 μm mesh size and passed through a bran finisher fitting a 125 μm mesh to produce white flour.

The *sbeII* and WT control wheat bread rolls were produced using a straight-dough method (AACC Method 10-10.03) at the Quadram Institute (NHS QI Clinical Research Facility, Norwich, UK). The formulation of each bread was adjusted based on the flour's starch content so that both provided the same total starch (∼75 g). Briefly, flour (1217.7 g and 1201.4 g for *sbeII* and WT control, respectively), yeast (35.6 g and 33.9 g for *sbeII* and WT control), sugar (44.5 g and 42.4 g for *sbeII* and WT control), salt (22.3 g and 21.2 g for *sbeII* and WT control), shortening (40.7 g and 38.8 for *sbeII* and WT control), and water (867.2 g and 781.6 g for *sbeII* and WT control) were mixed at low speed for 3 min and kneaded for 5 min at increasing speed, in a heavy-duty planetary mixer with hook attachment (model 5KSM7591XBSM, Kitchen Aid, Antwerp, BE). Dough was fermented 2 h at 21 °C then portioned into rolls and proofed for 15 min at 38 °C with 100% relative humidity, then baked using a combination oven (Rational Self Cooking Centre, Luton, UK) for 15 min at 185 °C (40% humidity for 10 min and 10% humidity for the last 5 min). Baked rolls reached ∼95 °C core temperature. After 2 h cooling at 21 °C, breads were packed in resealable, opaque polyethylene bags (3 mm thickness) and stored at −20 °C. Breads were produced in four batches of 12 rolls (48 rolls per type of flour), under identical conditions and paired by their baking position in the oven. Rolls were thawed for 16 h (overnight) and either served to human study participants as part of their breakfast meal or used for *in vitro* analyses. Breads were consumed within three months of manufacture.

### Bread characteristics and *in vitro* amylolysis

#### Nutrient composition and microbiological safety

Two pairs of bread rolls (*sbeII* and WT control) were randomly selected for proximate nutritional analysis and microbiological safety testing before the start of the study. These were performed by UKAS accredited testing at ALS Laboratories Ltd, Chatteris, Cambridgeshire, UK, as described by Bajka *et al.* (2021).^20^ Bread rolls were deemed safe for human consumption; details can be found in ESI Table 1.†

#### Starch characteristics

The proportion of digestible and resistant starch in flour were determined using a ‘Total starch kit’ (KTSTA-100A KOH format, AOAC 996.11, Megazyme International, Wicklow, Ireland), *n* = 8. Starch isolation and apparent amylose determination (*n* = 3) were carried out as described previously, Corrado *et al.* 2020.^14^

Five pairs of each bread type were randomly selected for *in vitro* analysis of starch characteristics. From each roll, a minimum of three technical replicates were sampled for analysis, as specified below. Rolls were left to thaw in their packaging at room temperature (∼21 °C) for 16 h, then were blended in a food processor (Kenwood CH 180 Mini chopper) for 50 seconds and sieved to obtain a 1 mm fraction, used for moisture determination and *in vitro* amylolysis assay, and a 500 μm fraction, used to measure digestible and resistant starch. These fractions were weighed into dry-tared aluminium pans in triplicates (technical replicates) for moisture determination, into 2 mL safe lock tubes (STARLAB (UK), Ltd) in quadruplicates (technical replicates) for starch measurements, and into 15 mL Corning centrifuge tubes (Merck KGaA, Darmstadt, DE) in triplicates (technical replicates) for amylolysis analysis. Digestible and resistant proportions of starch were measured using the reagents from ‘Total starch kit’ and ‘Resistant starch kit’ K-RSTAR (AOAC 2002.02, Megazyme International, Wicklow, Ireland), using a small-scale version of the method, as described by Edwards *et al.*, 2015.^21^ Moisture was determined using the AACC (44-15A) air oven method, one stage procedure.

#### *In vitro* starch susceptibility to amylase

Starch susceptibility to amylase digestion (amylolysis) was determined after five months of storage using an established amylolysis assay method based on enzyme-kinetic principles, on five bread rolls per type, three technical replicates. Full details of this method, which involves incubation of starch-rich food material with amylase, quantification of amylolysis products by PAHBAH, and calculation of starch digestibility indices, were recently published.^22^ Briefly, products of amylolysis were collected after 0, 3, 6, 9, 12, 15, 18, 21, 25, 30, 45, 60, 75, 90 min of incubation with porcine pancreatic α-amylase, as in a previous study on *sbeII* semolina pudding,^14^ and reducing sugars (maltose) were then quantified by the ‘PAHBAH’ (*p*-hydroxybenzoic acid hydrazide) assay. The resulting amylolysis curves were then baseline corrected by subtracting the value at *t* = 0 min (*Y*_0_) from each replicate. Starch digestibility was expressed as percentage of starch digested *C*_*t*_, and *C*_90_ representing extent of digestion after 90 min of incubation with α-amylase. Values for the first-order rate constant, *k* and endpoint, *C*_*∞*_,^23^ were obtained using a non-linear regression model to fit a first order equation *C*_*t*_ = *C*_*∞*_ (1 − *e*^−*kt*^) to the experimental data, using the *stats* package in Rstudio.^24,25^ Parameters were compared using independent groups *t*-tests, values reported throughout are means ± SEMs (*n* = 5) unless otherwise specified.

#### Bread roll quality assessment

Four pairs of bread rolls (*sbeII* and WT control) were randomly selected for quality assessment which included specific volume, crumb and crust colour, and texture. The rolls were left to thaw in their packaging at room temperature (∼21 °C) for 16 h. Bulk density (g cm^−3^) was determined using the rapeseed displacement method (AACC method 10-05.01); a metal block (14 × 6.3 × 5 cm) was used as the standard. Mass was determined immediately prior to rapeseed displacement measures on an OHaus (Model Explorer, NJ, USA) scale to 0.05 g precision. The specific bread volume (cm^3^ g^−1^) was determined as the volume/weight ratio of baked breads.

Crumb texture and bread colour were measured on crust and crumb. Texture was measured instrumentally using a ‘two-bite test’ (*n* = 4) on a TA-XT2 Texture Analyser (Stable Micro Systems, Godalming, UK),^20^ equipped with a five kg load cell using a modified AACC method 74–09. The Texture Analyser was equipped with a 50 mm diameter compression plate (P50); a uniaxial compression with crosshead speed of 100 mm min^−1^ was applied to 25 × 25 × 25 mm samples to mimic mastication, with crumb hardness corresponding to the force (*N*) required for 40% compression. Exponent (version 6.0, Stable Micro Systems, Godalming, UK) software for texture profile analysis was used to assess the following texture parameters: *hardness, springiness, cohesiveness, gumminess, chewiness and resilience*. Four independent measurements of colour were taken for each bread roll type, using a ‘Gretag Macbeth’ twenty-four patch Colour Checker as reference.^26^*Hue angle* and *Chroma* were calculated using the L*, a*, b* parameters.^27^ Parameters were compared using independent groups *t*-tests, values reported throughout are means ± SEMs (*n* = 4) unless otherwise specified.

### Acute postprandial intervention study

#### Ethical review

The REST study protocol procedures received favourable ethical opinion by the Health Research Authority England (South Cambridge Ethics Committee, REC reference 19/EE/0260, IRAS 262271) and was registered with ClinicalTrials.gov (NCT04197726). The study was also approved by the Human Research Governance Committee of the Quadram Institute and by the Department of Research and Development (R&D) of the Norfolk and Norwich University Hospitals NHS Foundation Trust (NNUH, reference 125-07-19). All participants provided written informed consent to take part in this study. All participants’ data were stored in accordance with the General Data Protection Regulation 2018 and biological samples were handled, stored, transported, and disposed of in accordance with the Human Tissue Act (2004).

#### Study design

We conducted a two arm two period double-blind randomised cross-over study comparing the effect of *sbeII* bread and WT control bread on glycaemic response, measured in capillary blood by FP test and in IF by CGM.

Enrolled participants were randomised in blocks of four stratified by sex to complete 3 visits: Visit 1, to apply the CGM sensors, followed by Visit 2 and Visit 3, where participants consumed either *sbeII* or WT control bread on consecutive occasions, in randomised order, with a minimum of four days washout period between visits with no dietary restriction. During intervention visits, capillary blood was obtained by fingerprick test to determine glycaemic response and in parallel, by CGM to measure glycaemic response in IF. The effect of *sbeII* bread consumption on satiety was determined using a visual analogue scale questionnaire (VAS)^28^ before and after bread consumption as well as changes in energy intake during the subsequent meal (satiety challenge).

Between interventions, participants completed a non-consecutive three-day weighted food diary, on two weekdays and one weekend day, to capture habitual diet. The study lasted approximately one month from screening to follow up.

#### A priori power calculation

The power calculation was carried out using an R core function, power.t.test.^24^ It was hypothesised that *sbeII* bread, with lower susceptibility to hydrolysis *in vitro* than the WT control, would induce a lower glucose response compared to the WT control *in vivo* (iAUC 0-120). A sample size of 19 was required to detect a 20% relative difference in iAUC difference with 80% power, assuming a 28.7 mmoL L^−1^ min^−1^ SD between pairs as observed in a previous study by Rosén *et al.* 2009.^29^ Planned sample size was increased to 25 to account for potential dropouts. Ultimately, owing to COVID restrictions, only eight participants could complete the study.

#### Study participants

Participants were recruited within 40 miles of the Norwich Research Park, Norfolk, UK. Participants meeting the following criteria were deemed eligible to take part: age 18–65 years, BMI 18–25 kg m^−2^, fasting glucose < 6.1 mmoL L^−1^, HbA1_c_ < 42 mmoL L^−1^, blood pressure < 160/100 mmHg. Participants were excluded if they were smokers, allergic or intolerant to the study foods or to adhesives, were alcohol or substance abusers or had insulin-dependent or non-insulin dependent diabetes, gastrointestinal disorder, anaemia, cardiovascular disease, certain cancers, or unstable body weight during the past 3 months. Women who were pregnant, lactating, or had given birth during the last 12 months were also excluded.

#### Study visits

The study consisted of three visits.

During Visit 1, participants applied two CGM *Freestyle Libre* sensors (Abbott Diabetes Care, Alameda, CA) to record IF glucose continuously for 14 days. They then observed a two-day CGM calibration period during which no interventions or restrictions were applied. Then, they completed Visit 2 and Visit 3 as follows: after a standardised evening meal and ∼12 h fast, baseline measurements of capillary blood glucose and satiety were taken while CGM sensors recorded IF glucose concentrations. They were then given the intervention meal as their first meal of the day (breakfast), either *sbeII* or WT control bread roll, after which glycaemic, sensory (palatability) and satiety responses (VAS) were measured over 3.5 h. Four hours after breakfast, a lunch meal was provided *ad libitum* as satiety challenge. At the end of the satiety challenge, the visit was considered completed and participants left the Clinical Research Facility of the Quadram Institute. A follow up questionnaire was completed the day after Visit 3 to capture participants’ experience wearing and using CGM devices.

#### Study meals

The standard evening meal consumed the night before Visit 2 and 3 consisted of a ready meal chosen from a pre-selected list of ready meals with similar nutrient composition. The ready meals contained ∼365 kcal, ∼39 g available carbohydrate, ∼8 g sugars, ∼8 g dietary fibre, ∼17 g protein, based on the nutrient declaration on food packaging. Only water was allowed between the evening meal and the intervention breakfast.

The intervention breakfast consisted of one bread roll providing ∼75 g of total starch, either *sbeII* or WT control, 10 g of low-fat spread (Flora Dairy Free) and approximately 250 mL of water to drink. The liquid component of the breakfast meal was standardised by adjusting the water drink to the water content of each bread. The total water content each meal was approximately 317 g, the average serving size of *sbeII* breads was 153.19 g ± 0.49 g (fresh weight) and the average size of WT control breads was 147.28 g ± 0.73 g (fresh weight), (mean ± SEMs, *n* = 8).

The lunch meal (satiety challenge) served *ad libitum* was made of portions of 80 g white rice, served with either ‘Option A’ 85 g of tomato and basil pasta sauce and 7 g of parmesan cheese or ‘Option B’ 90 g of beef Bolognese pasta sauce, and was served with 250 mL of water. Both sauce options had a similar nutrient composition and provided approximately 177.2 kcal ± 0.7 kcal, 27.3 g ± 0.4 g carbohydrate of which, 4.6 g ± 0.9 g sugars, 1.2 g ± 0.2 g fibre, 6.9 g ± 0.8 g protein per serving (average of two options ± SD, calculated from the nutrient declaration on food packaging). Participants were asked in advance to choose from sauce option A and B and consumed the same lunch meal during both visits.

#### Satiety challenge: *ad libitum* lunch

Four hours after consuming the intervention meal, participants consumed an *ad libitum* lunch to determine the effect of the intervention on subsequent energy intake (kcal). New bowls of rice with the sauce of choice (one portion) were provided at specific times depending on the speed of consumption of each participant (approximately every 3–10 min), to ensure that warm food was served and that finishing a bowl did not act as a cue to stop eating. Participants were instructed to eat until they were “comfortably satisfied”. Satiety changes were estimated based on VAS responses following bread consumption and the *total energy intake*, measured as the ‘kcal’, during the subsequent lunch *ad libitum*.

### Data and statistical analysis

#### Analysis of glycaemic response measures

Glucose concentrations measured in capillary blood by FP and in IF by CGM at pre-selected time points were used to compare the postprandial glycaemic responses to *sbeII* and WT wheat bread rolls.

Baseline fasting glucose was obtained 15, 10 and 5 min before the breakfast intervention meal by FP and at 30, 15 and 0 min before the breakfast intervention meal by CGM. The average of these three measurements was used as T0.

Intervention meals were consumed within 20 min, followed by ten pricks to measure capillary blood glucose concentrations, every 15 min for 3.5 h while glucose concentration in IF was recorded continuously every 15 min by the two sensors worn by each participant. Data up to 3.5 h was collected in order to capture the end of the postprandial response (return to baseline) to these study foods.

The glycaemic response to breads (iAUC) was calculated geometrically between 0 and 210 min (primary outcome), by applying the trapezoid rule to the area over the fasting glucose baseline and under the curve, as recommended by Brouns *et al.*^30^ Average glucose peak and glucose dip were calculated as the maximum (peak) and minimum (dip) glucose concentrations achieved after each meal; for capillary glucose this was calculated between 15 and 210 min, for IF glucose, between 15 and 240 min. Time to peak was calculated as the time to reach the maximum glucose concentration following the meal.

#### Statistical models

Capillary and IF glucose iAUCs were compared across groups using a mixed-effects model using the lmer package v. 3.1.2^31^ in Rstudio, with bread type as fixed effect and a random intercept per participant. The model used to obtain the IF effect estimates also included the visit nested within participant, as an additional random intercept to account for having data from two CGM sensors. Estimates of effect were obtained with Satterthwaite approximations for degrees of freedom, using the emmeans package v. 1.4.5^32^ in Rstudio and Pearson pairwise correlation (within participant, between sensors) was determined using the *stats* package previously mentioned.

Datasets were curated using package reshape v. 1.4.3, graphs and plots were made using ggplot2 v. 3.3.1 and ggpmisc v. 0.3.5^33–35^ in Rstudio.

#### Analysis of satiety and sensory measures

VAS questionnaires were completed before the meal (T0), and at 30, 90, 180 and 270 min post-prandially to determine changes in *hunger, fullness, desire to eat*, and *prospective consumption*. The scales consisted of horizontal lines 100 mm long, anchored by “not at all” and “extremely” at opposite ends. Satiety VAS questionnaires were scanned using ImageJ (NIH, USA),^36^ and an appetite score was calculated as follows: appetite score = [desire to eat + hunger + (100 − fullness) + prospective consumption]/4 as described by Anderson *et al.* (2010).^37^

## Results

### Flour characteristics

The *sbell* and WT control flours had similar total starch (73.9 ± 1.2 g *vs*. 74.94 ± 0.8 g per 100 g of flour, means ± SEMs, *n* = 8). Apparent amylose (means ± SEMs, *n* = 3) was higher in *sbell* starch (39 ± 1.1% of total starch) than in WT control starch (25.7 ± 0.7% of total starch), hence the proportion of resistant starch (means ± SEMs, *n* = 8) was much higher in *sbell* flour (6.4 ± 0.5% of total starch) compared to WT control (0.6 ± 0.1% of total starch). Indicators of performance in the field and extraction rate can be found in ESI Table 2.†

### Bread characteristics

**Fig. 1:**
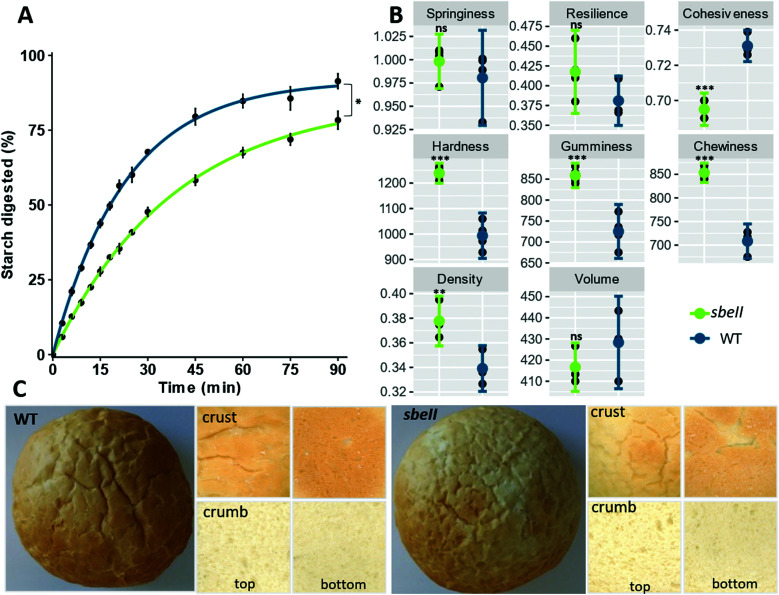
*In vitro* characteristics of *sbeII* (green) and WT (blue) bread rolls after 5 months of storage at −20 °C. (A) Starch amylolysis; each experimental data point represents the mean value from the analysis of five independent samples with error bars = ± SEMs, *n* = 5. Experimental data are shown by fitting a first-order equation based on the *k* and *C*_∞_ values obtained from a non-linear regression model. (B) Bread quality parameters, mean ± SEMs, *n* = 4; bread crumb texture analysis on Texture Analyser showing springiness, cohesiveness, resilience (%), and hardness (Newtons), gumminess (AU) chewiness (Newtons per s); bread volume and density (bulk density). (C) Images of bread rolls as whole, crust and crumb.

Breads were formulated to deliver approximately 75 g of total starch per serving. Nutrient composition, indicators of *in vitro* amylolysis and starch characteristics of bread rolls are reported in Table 1. Starch in *sbeII* bread was less susceptible to amylolysis compared to the WT control (*C*_90_ mean difference = 13.03% of starch digested, 95%CI [1.91, 24.15], independent groups *t*-test, *p*-value = 0.02). First order equations fit starch digestion curves well, Table 1. The *sbeII* bread was characterized by a lower starch breakdown after 90 min of hydrolysis and a lower digestion rate (*k*), Fig. 1A.

Compositions of the morning intervention meal with either *sbeII* or WT control bread. Bread and starch characteristics measured at the end of recruitment (5 months after production and storage at −20 °C)

**Table:** 

Nutrients per serving	WT control bread meal	*sbeII* bread meal
Energy^a^ (kcal)	438.7	430.8
Energy (kJ)	1854.9	1815.7
Protein^b^ (g)	12.15	12.96
Total fat^c^ (g)	11	11.54
Saturated fat (g)	2.92	3.07
Poly-unsaturated fat (g)	3.54	3.77
Ash	2.19	2.29
Available carbohydrates^d^ (g)	70.9	65.1
Total sugars (g)	2.92	3.06
Fibre AOAC^e^ (g)	4.53	7.5

**Table:** 

Bread characteristics	WT bread	*sbeII* bread
Bread roll (g)	146.24 ± 2.55	153.24 ± 0.38***
Moisture (%)	38.59 ± 0.17	42.13 ± 1.02***
Total starch (g)	74.9	74.9
*Digestible starch (%)*	96.5 ± 0.2	91.8 ± 0.8***
*Resistant starch (%)*	3.5 ± 0.1	8.2 ± 0.2***

**Table:** 

*In vitro* amylolysis	WT bread	*sbeII* bread
*C*_90_ (%)	91.3 ± 2.9	78.3 ± 3.7*
*C*_∞_ (%)	91.6	85.4
*k* (min^−1^)	0.048	0.030
*r* ^2^	0.99	0.99
Sum of squares	1706	767

a Calculated using standard energy conversion factors (EC 2008/100 and 90/496).

b Nitrogen-to-protein conversion factor = 6.25.

c Determined by NMR.

d Determined by ion exchange chromatography, calculated by difference.

e AOAC method 985.29 (‘Prosky method’), which includes RS type 3 (retrograded starch).

*Hardness, gumminess, chewiness and cohesiveness* were significantly higher in *sbeII* bread rolls compared to WT control breads while there were no differences in *springiness* and in *resilience* between bread types (Fig. 1B.1 and B.2). The bulk density of *sbeII* bread was greater than the WT control but no differences in volume were detected (Fig. 1B.3 and B.4). Crust colour varied between *sbeII* and WT breads with a darker colour and a matte finish for the *sbeII* bread crust, compared to the WT control (Fig. 1C), ESI Table 3.†

### Acute postprandial intervention study

**Fig. 2:**
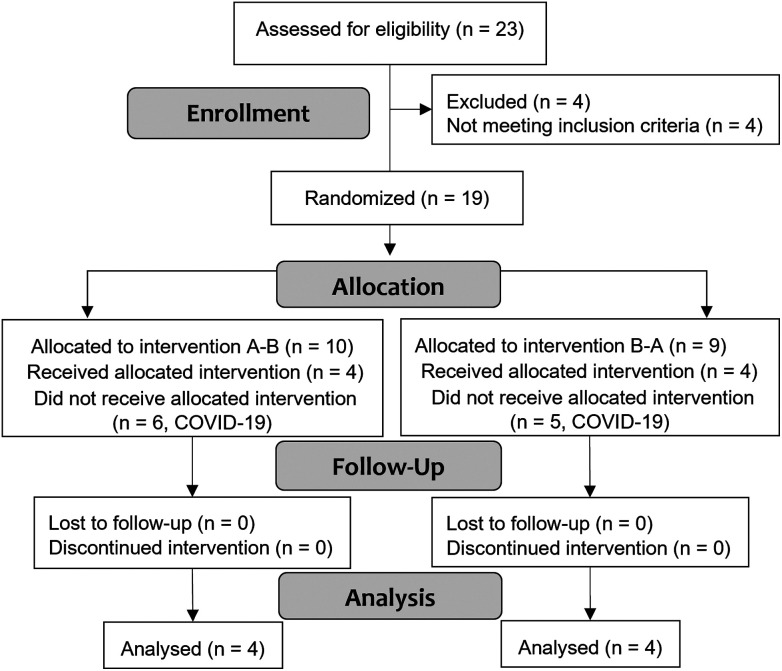
CONSORT diagram of the REST study. Interventions were blinded to the participants and the study team using an allocation concealment. At the end of recruitment, breads were grouped as ‘A’ or ‘B’ to allow the study team to complete the data analysis. Researchers were unblinded once the data analysis was completed. Two participants lost one sensor after intervention 1 and completed intervention 2 with one sensor only. Two participants lost one sensor before intervention 1 and had it replaced.

Only 8 out of 19 enrolled participants completed the study due to the COVID-19 outbreak and consequent lockdown (Fig. 2). Their characteristics are reported in Table 2. All other baseline measurements are reported in ESI Table 4.† The average energy intake recorded by participants during the three-day food diary was within the UK dietary recommendations^38^ and their carbohydrate intake was approximately 41% of their total kcal intake (men and women), predominantly derived from starch Table 2. More details about the average dietary intake of study participants can be found in ESI Table 5.†

Screening characteristics and habitual dietary intake of study cohort (inclusion/exclusion criteria)

**Table:** 

Characteristics	Mean	SD	Min	Max
Age (years)	33	13.6	23	58
Height (m)	1.73	8.6	1.60	1.90
Weight (kg)	69.8	10.4	58.1	91.9
BMI (kg m^−2^)	23.2	1.7	20.7	25.4
Venous fasting glucose (mmoL L^−1^)	4.4	0.2	4.1	4.7
HbA1c (mmol mol^−1^)	34.4	2.0	31.0	36.0

**Table:** 

Three-days average intake	Men	Women
Energy (kcal per day)	2780.5 ± 735.9	1989 ± 430.9
Starch (g per day)	187.6 ± 49	136.8 g ± 39.8
Non-starch polysaccharide (g per day)	24.9 g ± 3.3	18.7 g ± 3.1

#### Glycaemic response

Participants required approximately 8–10 min to consume the intervention meals. The estimates of effects of *sbeII* and WT control breads on postprandial glucose responses are reported in Table 3.

Glucose responses measured by fingerprick test (FP) and continuous glucose monitoring system (CGM) and other indicators of glycaemic response to intervention meals. IF values are reported as mean of the 8 participants that completed the study, average of two CGM sensor readings per participant

**Table:** 

iAUC (mmoL L^−1^ min^−1^)	WT control bread	*sbeII* bread	*Treatment effect*
FP_0–120_	140 [109.1, 172]	118 [86.2, 149]	−22.9 [−59.5, 13.6]
CGM_0–120_	128 [93.5, 162]	109 [74.5, 143]	−19.2 [−67.5, 29.1]
FP_0–210_	182 [146, 219]	157 [121, 194]	−25.1 [−82.3, 32.2]
CGM_0–210_	136 [80.3, 191]	103 [47.5, 158]	−32.9 [−111, 45.1]

**Table:** 

Bread type	Method	Fasting glucose (mmoL L^−1^)	Glucose peak (mmoL L^−1^)	Glucose dip (mmoL L^−1^)	Time to peak (min)	Time to baseline (min)
*sbeII*	FP	5.1 ± 0.06	7.1 ± 0.25	4.9 ± 0.08	39.4 ± 3.9	185 ± 9.2
	CGM	4.7 ± 0.05	6.4 ± 0.29	4.5 ± 0.16	53.0 ± 2.9	210 ± 12.6
WT control	FP	5.03 ± 0.07	7.3 ± 0.24	4.8 ± 0.14	67.5 ± 19.4	175 ± 12
	CGM	4.6 ± 0.1	6.7 ± 0.36	4.2 ± 0.25	49.3 ± 1.9	184 ± 10.8

**Fig. 3:**
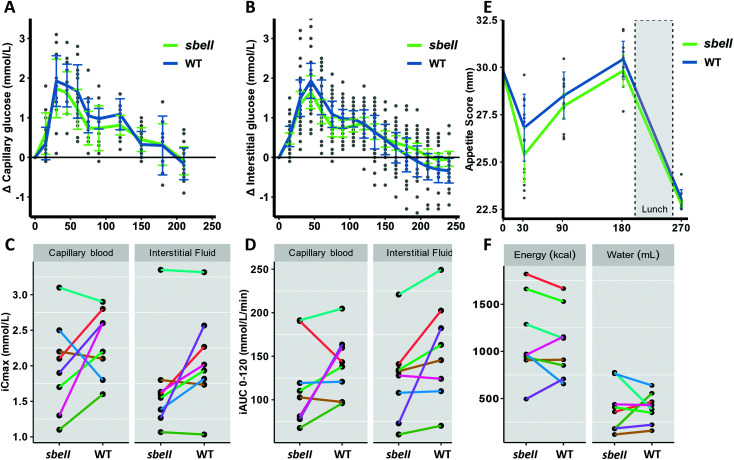
Post-prandial glucose concentrations. Incremental glucose concentrations measured in capillary blood (A) and IF (B). Datapoints represent individual glucose values, curves are mean glucose profile (*n* = 8) and error bars = ± SEMs. C. Incremental maximum concentration of glucose (iCmax) in capillary and IF. (D) Individual incremental glucose over time detected in capillary blood and IF (iAUC_0–120_). (E) Change in appetite score from fasting until after the *ad libitum* lunch (satiety challenge), score calculated as [desire to eat + hunger + (100 − fullness) + prospective consumption]/4, *n* = 8 and error bars = ± SEMs. (F) Energy and water intake during satiety challenge following the intervention meals (*n* = 8).

Glycaemic response (iAUC) to *sbell* bread was 22.9 mmoL L^−1^ min^−1^, 95% CI [−13.6, 59.5], lower than control bread (corresponding to an average 15.7% reduction following *sbeII* bread compared to WT control bread). Similarly, the effect size measured in IF by CGM was 19.2 mmoL L^−1^ min^−1^ (14.8% difference between *sbeII* and WT control breads). While these estimates from only 8 participants are not statistically significant, they are consistent with the *in vitro* findings and our anticipated effect size of 20% difference between groups. Participants’ fasting glucose concentrations measured in capillary blood and IF were within normal range and their fasting glucose values did not vary significantly between the two visits (FP fasting glucose *p*-value = 0.58, IF fasting glucose *p*-value = 0.62, paired *t*-test). The overall pattern of postprandial capillary blood glucose was similar after the consumption of *sbeII* bread and WT control (Fig. 3A and B). Fig. 3C shows the incremental glucose peak (iCmax) and Fig. 3D shows the average capillary glucose responses (iAUC_0–120_). Other postprandial capillary glucose indicators are reported in Table 3. Time to peak calculation from the start of the test showed that the IF measurements lagged 11 min compared to capillary blood glucose.

#### Satiety responses

Participants indicated that their ‘desire to eat’ and ‘hunger’ were similar after consuming *sbeII* and WT control bread, as measured by the satiety VAS questionnaires, Fig. 3E. There is some evidence of increased ‘fullness’ up to 30 min after consuming *sbeII* bread (paired *t*-test, mean difference = 2.16, 95% CI [0.65, 3.6], *p*-value = 0.01). Plots showing individual satiety responses can be found in ESI Fig. 1.† The increased ‘fullness’ appeared to be temporary and did not seem to affect the following meal (lunch), as subsequent energy and water intake *ad libitum* (lunch satiety challenge) did not differ following the two intervention meals (energy intake = 1131.04 ± 154 kcal and 1076.72 ± 130 after consuming *sbeII* and WT control breads respectively, means ± SEM, *n* = 8, and water intake = 402 mL ± 90 mL and 400 mL ± 56 mL after *sbeII* and WT control breads respectively, means ± SEM, *n* = 8), Fig. 3F.

#### Comparing FP and CGM measures of postprandial glycaemia

Each participant wore two identical CGM sensors for all intervention visits on the same arm. Two participants lost one sensor after intervention 1 and completed intervention 2 with one sensor only. Two other participants lost one sensor before intervention 1 and had it replaced. Based on the follow up questionnaire completed by the study participants, the experience of wearing two CGM sensors was described as “quick” and “easy”, some participants found the application slightly painful “like a jab” however, most described them “comfortable” to wear and “interesting”.

Intra-individual variation (CV%) between the two sensors used in this study by each participant ranged 0–17% during postprandial breakfast phase and 0–22.7% during the postprandial lunch phase. There was a strong correlation between the two CGM sensors worn by each participant (*r* = 0.91); on average, there was a 10.5 mmol L^−1^ min^−1^ difference between sensors’ measurements of glycaemic response (iAUC 0–120) within participant. We observed a 5.25 mmol L^−1^ min^−1^, 95% CI [−11.3, 21.8] difference between sensors after consuming *sbeII* bread (*n* = 8) and 17.6 mmol L^−1^ min^−1^, 95% CI [−5.73, 40.9] after WT bread (*n* = 6). Glucose response curves are shown in ESI Fig. 3.†

**Fig. 4:**
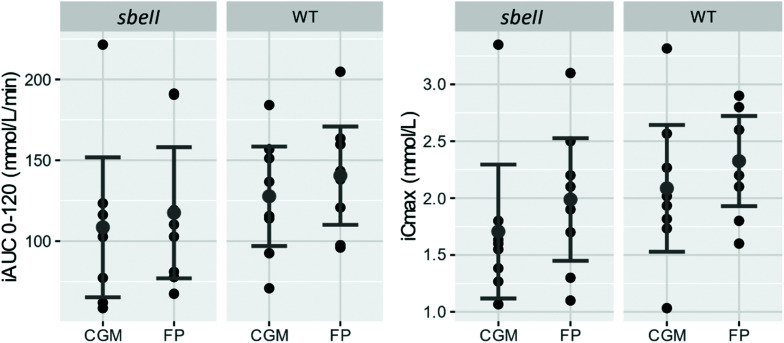
iAUC and iCmax measured by CGM in IF and in capillary blood by FP after consuming *sbeII* and WT control bread, respectively. Data points represent individual responses (black), the mean of eight subjects in grey with error bars = ± SEMs.

Overall, the glucose response measured by FP and by CGM was similar (iAUC and iCmax), Fig. 4.

## Discussion

This study investigated the use of a *sbeII* mutant wheat with a higher amylose proportion for production of slowly digested and/or low glycaemic bread products, compared to conventional wheat starch. Bread rolls made from *sbeII* mutant wheat contained more RS and showed lower susceptibility to amylase digestion that the WT control, which is consistent with the hypothesis that the presence of mutations in *sbeII* genes lead to wheat foods with increased starch resistance to digestion.^14^

Previous studies measuring glycaemic potency of high-amylose food products reported conflicting results. Often, this is derived from inconsistent carbohydrate content across test meals; Behall *et al.* in 2002^39^ presented a very good overview of the different studies that compared glycaemic responses to high and low amylose food products and showed that different methods used to determine the RS and dietary fibre can contribute to the variability in glycaemic measurements. In this study, we report amylose and RS portion of starch in bread rolls, as well as starch resistance to digestion measured using a single-enzyme system that allowed us to determine the rate and extent of starch digestion, previously shown to correlate with glycaemic index measured *in vivo.*^22^

Here, we showed that the predicted difference in glycaemic response between *sbeII* and WT control breads from *in vitro* amylolysis experiments is consistent with the observed difference in glucose concentrations after consumption of *sbeII* and WT control breads, although our estimate of the effect *in vivo* is imprecise because only around one third of the planned sample could be completed.

Studies using novel wheat genotypes characterized by a high amylose content have consistently reported a lowering effect on postprandial glucose response. Previously, Belobrajdic *et al.*^16^ reported a lower glycaemic response to high amylose wheat bread measured in plasma glucose. The low glycaemic effect was likely due to the reduced amount of carbohydrate provided in the high-amylose bread compared to the control bread. In this study, we showed that *sbeII* bread has potential to elicit lower glycaemic response compared to a conventional white bread without changing the amount of starch provided.

Hallström *et al.* (2011)^43^ showed that bread made from a high-amylose wheat (38% of starch) induced 24% lower glycaemic responses than a control white bread (high-amylose bread iAUC 0–120 min = 185 ± 29.7 mmoL L^−1^ min^−1^, white control bread iAUC 0–120 min = 224 ± 27.3 mmoL L^−1^ min^−1^, mean ± SEM, *n* = 14) although all bread types in their study were baked at pumpernickel conditions (20 hours, 120 °C), which may have modulated starch resistance to enzymatic digestion. In the present study, the difference in starch susceptibility to amylase digestion between *sbeII* and WT control breads was observed using a shorter baking time (15 min) at higher temperature (185 °C) to produce white rolls, resembling the Chorleywood baking process.

It is noteworthy that these breads were stored at −20 °C for ∼2 months longer than those consumed by the human study participants. There is still a limited understanding of the effect of freeze–thaw treatment on starch digestibility in bread and glycaemia, however the largest effects of cold storage occur during the initial stages of freezing^40^ and further starch retrogradation effects thereafter would likely be minimal and negligible with regard to influencing postprandial glycaemia.^41^

A study by Sisson *et al.* in 2020^15^ showed that pasta made from *sbeIIa* mutant semolina (with 58% amylose starch) produced pasta with lower glycaemic index (compared to an isoglucidic WT control pasta) and acceptable quality, although the authors did report slightly reduced firmness, increased cooking loss and inferior colour compared to the control, likely to be linked to the large increase in amylose. The *sbeII* bread used in the present study presented minor differences in texture compared to the WT control but was deemed overall acceptable by participants.

The results presented in this study showed that the effect of bread on glycaemic response (iAUCs) were measured similarly in IF by CGM on the upper arm and in capillary blood by FP, therefore the conclusions based on iAUCs does not differ between the metrics. Measuring glucose responses in IF by CGM was found to be an effective and minimally invasive method to carry out intervention studies: sensor measurements are reliably taken every 15 min (this may vary depending on the type of sensor), allowing studies to collect larger glucose datasets compared to capillary glucose measured by FP, which is also more painful for study participants. Previous studies have shown a good agreement between measurements taken in blood and IF^42^ however, the IF measurements can vary depending on the type of device and the insertion site.^18^

Based on the results collected from eight participants, the effect size measured in this study in IF was consistent with the expected effect size based on *in vitro* amylolysis. The study was underpowered due to the COVID-19 outbreak that forced us to stop all study activities ahead of time. Nevertheless, the results from the human study suggested that bread made with *sbeII* mutant wheat may lead to lower postprandial IF glycaemic responses.

Overall, this study showed that the small increases in amylose content in the *sbeII* mutant wheat raw material can be used to produce *sbeII* wheat-based foods with lower starch susceptibility to hydrolysis, without substantially compromising (bread) texture or appearance, measured instrumentally. Future foods made from *sbeII* mutant wheat may provide an alternative to high glycaemic wheat-based staple foods, including white bread. Further acute and chronic intervention studies are needed to fully explore the potential use of *sbeII* mutant wheat to reduce the glycaemic potency of bread and other wheat-based foods.

## Abbreviations

IF: Interstitial fluid
FP: Fingerprick
CGM: Continuous glucose monitoring
*sbeII*
: *Starch branching enzyme II*
WT: Wild-type
RS: Resistant starch
iAUC: Incremental area under the curve

## Authors contribution

Marina Corrado: Conceptualization, investigation, methodology, project administration, data curation and formal analysis, writing – original draft preparation, writing – review & editing, Jennifer Ahn-Jarvis: investigation, methodology, writing – review & editing, Brendan Fahy: investigation, writing – review & editing, George M. Savva: methodology, data curation, formal analysis, Cathrina H. Edwards: conceptualization, methodology, supervision, writing – review & editing, Brittany A. Hazard: conceptualization, funding acquisition, methodology, supervision, writing – review & editing.

## Conflicts of interest

The authors declare no conflicts of interest.

## Supplementary Material

FO-013-D1FO03085J-s001

FO-013-D1FO03085J-s002
