# Supplementary material for: Effect of high-amylose starch branching enzyme II wheat mutants on starch digestibility in bread, product quality, postprandial satiety and glycaemic response
Source: Food Funct. 2022 Jan 26;13(3):1617–27. doi: 10.1039/d1fo03085j (PMC8820503; doi:10.1039/d1fo03085j)
Supplement: FO-013-D1FO03085J-s001 [file FO-013-D1FO03085J-s001.pdf]

Electronic Supplementary Material (ESI) for Food & Function.
This journal is © The Royal Society of Chemistry 2022

Effect of high-amylose *starch branching enzyme II* wheat mutants on starch digestibility in bread, product quality, postprandial satiety and glycaemic response.

Marina Corrado[i], Jennifer H. Ahn-Jarvis[1], Brendan Fahy[ii], George M. Savva[1], Cathrina H. Edwards[1 ¥ *] and Brittany A. Hazard[1 2 ¥]

Source data

Table 1. Capillary glucose concentrations REST study

Capillary glucose concentrations after consuming *sbeII* bread											
**Participant ID**	Time (min)										
	0	15	30	45	60	75	90	120	150	180	210
1	4.9	6.7	6.7	6.8	7	6.4	6.7	5.8	5.9	6.3	5.3
2	4.9	4.9	6.7	7.1	6	5.8	5.2	5.3	5.1	5.8	5
3	4.8	4.8	4.9	5.9	5.7	5.2	5.8	5.3	5.5	5.3	5.4
4	5.2	5.4	6.9	6.9	6.1	6	6.1	5.9	5.5	4.8	4.8
5	5.2	5.9	8.3	7.7	7.2	6.6	6.7	6	5.2	5	4.8
6	5.1	6.3	7.6	6.2	6.6	5.5	5.2	6.2	5.2	5	4.8
7	4.9	5.3	6.6	6.8	5.3	5	4.8	5.7	5.7	5.5	4.8
8	5.9	6.4	7.1	6.5	6.7	6.3	6.3	7.2	6.4	5.8	5.3
**Capillary glucose concentrations after consuming WT bread**											
**Participant ID**	Time (min)										
	0	15	30	45	60	75	90	120	150	180	210
1	5.4	5.3	7.4	6.5	8.2	6.5	6.4	6.5	5.3	5.2	5.2
2	4.7	5	6.2	6.3	5.2	5.2	5.3	5.9	5.3	6.8	5.4
3	4.8	4.9	5.4	5.9	6.2	5.5	5.4	6.4	5.4	5.2	5
4	5.6	5.5	7	7.4	7.8	6.5	6.5	7.2	5.7	5.2	5.2
5	5.1	5.2	8	8	7.7	7.3	6.6	5.8	5	4.4	4.2
6	4.8	6	6.6	6.2	6	5.7	5.7	5	4.8	4.7	4.3
7	4.6	4.9	7.2	7	6.2	5.8	5.6	5.9	5.2	5	4.5
8	5.3	6.3	7.9	7.5	6.3	6.2	6.6	6.3	6.2	6.2	5.2

Time = 0 is the average of three measurements collected at -15 min, -10 min, -5 min (before starting to eat the test meal)

Table 2. Interstitial glucose concentrations REST study (sbeII bread)

Interstitial glucose concentrations after consuming *sbeII* bread																		
**Participant ID**	Sensor	Time (min)																
	A	**0**	**15**	**30**	**45**	**60**	**75**	**90**	**105**	**120**	**135**	**150**	**165**	**180**	**195**	**210**	**225**	**240**
1	A	5.2	5.4	6.3	6.9	6.9	6.6	6.2	6.1	6.1	5.9	5.6	5.6	5.6	5.6	5.4	5.2	5.1
2	A	4.3	4.4	4.9	5.8	5.8	5.3	5	5	5.1	5.2	5.2	4.9	4.8	4.9	4.8	4.6	4.4
3	A	4.1	4.3	4.7	5.2	5.2	4.7	4.4	4.6	4.7	4.8	4.8	5	4.9	4.7	4.7	4.8	4.7
4	A	4.4	4.7	5.6	6.1	5.8	5.3	5.3	5.6	5.6	5.2	4.9	4.7	4.6	4.4	4.3	4.2	4.1
5	A	4.7	4.6	5.7	7.6	7.9	7.1	6.7	6.5	5.9	4.8	4.2	4.3	4.2	3.8	3.6	3.4	3.4
6	A	4.6	4.4	5	5.9	6.1	5.7	5.3	4.9	4.9	5.1	4.9	4.8	4.7	4.6	4.6	4.5	4.4
7	A	4.7	4.7	5.4	6	5.6	4.8	4.6	5	5.5	5.8	5.7	5.3	5.2	5	4.7	4.4	4.2
8	A	5.0	5.9	6.8	6.6	5.9	5.7	5.8	6.2	6.4	6.1	5.7	5.6	5.3	5.2	5.1	4.9	4.9
1	B	4.9	5.2	6	6.4	6.4	6.2	5.9	5.8	5.7	5.5	5.3	5.3	5.3	5.3	5.1	5.1	4.9
2	B	4.9	4.8	5.3	6.7	7	6.1	5.5	5.6	5.8	5.9	5.8	5.4	5.2	5.6	5.5	5.1	4.9
3	B	5.2	5.1	5.4	5.9	6.2	5.8	5.4	5.6	5.7	5.7	5.8	5.9	5.9	5.8	5.8	5.7	5.8
4	B	4.1	4.2	4.6	5.3	5.5	5.2	5.2	5.4	5.3	5.1	4.8	4.6	4.4	4.3	4.2	4	3.9
5	B	5.1	5.4	6.9	8.6	8.4	7.4	7.1	7	6.3	5.1	4.7	4.7	4.4	4.1	4	4.1	4.1
6	B	4.6	4.6	4.7	5.2	5.8	5.9	5.6	5.3	5.1	5	5.1	4.8	4.7	4.6	4.6	4.6	4.6
7	B	4.7	4.7	5	5.7	5.9	5.4	4.9	5	5.2	5.6	5.9	5.9	5.7	5.4	5.1	4.7	4.6
8	B	5.2	5.1	5.8	6.7	6.7	6	5.7	5.7	6.2	6.4	6.2	5.8	5.7	5.5	5.3	5.2	5.1

Time = 0 is the average of three measurements collected at -30 min, -15 min, 0 min (before starting to eat the test meal)

Table 3. Interstitial glucose concentrations REST study (WT bread)

Interstitial glucose concentrations after consuming WT bread																		
**Participant ID**	Sensor	Time (min)																
	A	**0**	**15**	**30**	**45**	**60**	**75**	**90**	**105**	**120**	**135**	**150**	**165**	**180**	**195**	**210**	**225**	**240**
1	A	n/a	n/a	n/a	n/a	n/a	n/a	n/a	n/a	n/a	n/a	n/a	n/a	n/a	n/a	n/a	n/a	n/a
2	A	n/a	n/a	n/a	n/a	n/a	n/a	n/a	n/a	n/a	n/a	n/a	n/a	n/a	n/a	n/a	n/a	n/a
3	A	4.4	4.7	5.1	5.4	5.3	5	5.1	5.2	5.1	4.9	4.8	4.8	4.7	4.6	4.7	5.1	5.3
4	A	4.8	5.2	6.1	6.9	6.8	6.3	6.1	6.3	6.6	6.2	5.6	5.1	4.9	4.7	4.6	4.4	4.4
5	A	5.4	5.7	7.6	8.8	7.7	6.8	6.8	6.4	6.1	5.5	5.1	5	5	4.9	4.8	4.8	4.8
6	A	5.0	5.4	6.4	6.9	6.1	5.6	5.8	5.8	5.6	5.1	4.8	4.7	4.4	4.6	4.8	4.9	4.8
7	A	4.6	4.9	6.3	7.4	6.8	5.7	5.4	5.9	6.1	6.1	5.8	5.4	5.2	4.8	4.4	4.2	4.1
8	A	5.0	5.2	6.4	7.2	6.4	5.6	5.7	5.9	5.8	5.9	6	6.1	6.1	5.6	4.8	4.4	4.3
1	B	3.2	3.3	4.6	5.5	5.4	4.9	4.4	4.4	4.4	4.1	3.6	3.3	3.2	3.2	3.1	2.9	2.7
2	B	4.9	4.7	5.1	5.9	6.6	6.1	5.4	5.7	6.1	6	5.8	5.6	5.5	5.9	5.9	5.4	5
3	B	5.1	5.1	5.4	5.9	6.2	5.8	5.6	5.7	5.8	5.7	5.6	5.4	5.3	5.2	5.1	5.4	5.7
4	B	3.2	3.2	3.6	4.6	5	4.5	4.1	4.2	4.5	4.5	3.8	3.4	3.2	3.1	2.9	2.8	2.8
5	B	5.5	6.1	7.9	8.7	7.4	6.8	6.8	6.4	6.2	5.5	5.1	5.2	5.2	5.1	4.9	4.9	5.1
6	B	4.7	4.9	5.7	6.4	6.3	5.8	5.6	5.5	5.4	5.2	4.8	4.6	4.4	4.3	4.3	4.4	4.6
7	B	4.8	4.8	5.6	6.8	7.1	6.3	5.6	5.7	6.1	6.1	6.1	5.7	5.3	5.1	4.8	4.6	4.3
8	B	5.0	5.3	6.3	6.8	6.2	5.7	5.7	5.9	5.9	5.9	6.1	6.2	6.2	5.7	4.9	4.5	4.3

Time = 0 is the average of three measurements collected at -30 min, -15 min, 0 min (before starting to eat the test meal)

[i] Food Innovation and Health, Quadram Institute Bioscience, Norwich Research Park, UK
[ii] Designing Future Wheat and Molecules from Nature, John Innes Centre, Norwich Research Park, UK
[¥] These authors contributed equally.
