# Supplementary material for: Effect of high-amylose starch branching enzyme II wheat mutants on starch digestibility in bread, product quality, postprandial satiety and glycaemic response
Source: Food Funct. 2022 Jan 26;13(3):1617–27. doi: 10.1039/d1fo03085j (PMC8820503; doi:10.1039/d1fo03085j)
Supplement: FO-013-D1FO03085J-s002 [file FO-013-D1FO03085J-s002.pdf]

Electronic Supplementary Material (ESI) for Food & Function.
This journal is © The Royal Society of Chemistry 2022

Effect of high-amylose *starch branching enzyme II* wheat mutants on starch digestibility in bread, product quality, postprandial satiety and glycaemic response.

Marina Corrado[1], Jennifer H. Ahn-Jarvis[1], Brendan Fahy[2], George M. Savva[1], Cathrina H. Edwards[1 ¥ *] and Brittany A. Hazard[1 2 ¥]

Online Supplementary Material

[1] Food Innovation and Health, Quadram Institute Bioscience, Norwich Research Park, UK
[2] Designing Future Wheat and Molecules from Nature, John Innes Centre, Norwich Research Park, UK
[¥] These authors contributed equally.

Table 1. Certificate of microbiology analysis by ALS Laboratories (UK) Limited - Sands Mill, Huddersfield Road, Mirfield, West Yorkshire, WF14 9DQ

Bread type	Method	Test	Result	Units
sbeII bread	ESGM-M300	Total Viable Count, 2 days	<10	cfu / g
	ESGM-M303	Enterobacteriaceae (presumptive)	<10	cfu / g
	ESGM-M304	Escherichia coli (β-Glucuronidase positive)	<10	cfu / g
	ESGM-M307	Coagulase positive Staphylococci	<20	cfu / g
	ESGM-M319	Bacillus cereus (presumptive)	<20	cfu / g
	ESGM-M310	Clostridium perfringens (presumptive)	<10	cfu / g
	ESGM-M308	Yeasts	<20	cfu / g
	ESGM-M308	Moulds	<20	cfu / g
	ESGM-M515	Salmonella sp. (ELISA)	Not Detected	/ 25g
WT control bread	ESGM-M300	Total Viable Count, 2 days	<10	cfu / g
	ESGM-M303	Enterobacteriaceae (presumptive)	<10	cfu / g
	ESGM-M304	Escherichia coli (β-Glucuronidase positive)	<10	cfu / g
	ESGM-M307	Coagulase positive Staphylococci	<20	cfu / g
	ESGM-M319	Bacillus cereus (presumptive)	<20	cfu / g
	ESGM-M310	Clostridium perfringens (presumptive)	<10	cfu / g
	ESGM-M308	Yeasts	<20	cfu / g
	ESGM-M308	Moulds	<20	cfu / g
	ESGM-M515	Salmonella sp. (ELISA)	Not Detected	/ 25g

The two genotypes were each sown in eight 6 m^2 plots in a randomised block design in in April 2018. Yield and thousand grain weight (TGW) are reported in Table 2 as indication of grain performance when grown in the UK. Kernel weight was determined using a Marvin Seed Analyser (Marvitech GmbH) with a sample of approximately 300 grains per plot. The harvested grains were stored in the field trial station until October 2018, when they were milled into refined flour by Campden BRI using a Bühler mill.

Table 2. Average kernel weight and yield of sbeIIa/b-AB, sbeIIa-D and WT control wheat. Milling extraction rate is reported here.

**Genotype**	**Sowing season**	**TGW (g)**	**Yield (tons/ha)**	**Milling extraction rate (%)**
sbeIIa/b-AB, *sbeIIa*-D	Spring	32.2 ± 0.54	3.5 ± 0.117	73.15 ± 0.30
WT control	Spring	33.8 ± 0.71	3.9 ± 0.16	77.97 ± 0.22

Means ± SEMs, n = 8.

Table 3. Bread crust colour analysis

**Bread type**	**L***	**a***	**b***	**Hue angle**	**Chroma**
WT control	78.2 ± 0.7	11.6 ± 0.6	40.7 ± 0.8	105.7 ± 0.7	42.4 ± 0.9
sbeII	83.7 ± 0.9*	6.7 ± 0.8*	35.4 ± 1.3	100.3 ± 1.1	36.1 ± 1.4

Mean ± SEMs, n = 4 bread rolls, 16 readings per bread roll (four on each roll side).

Parameter values that are significantly different in the *sbeII* bread compared to the WT control bread are indicated with an asterisk; *$P < 0.05$, **$P < 0.01$ and ***$P < 0.001$ (independent groups t-test).

Table 4. Baseline characteristics measured at screening of the 4 men and 4 women who consumed WT control and sbeII bread

**Characteristics**	**Mean**	**SD**	**Min**	**Max**
Systolic blood pressure (mmHg)	124	10.7	108	137
Diastolic blood pressure (mmHg)	81	6.1	69	88
Cholesterol (mmol/L)	4.8	0.9	3.1	6.2
HDLC (mmol/L)	1.5	0.4	0.8	2.1
LDLC (mmol/L)	2.9	0.8	1.6	3.8
Triacylglycerol (mmol/L)	0.8	0.3	0.6	1.5
Total bilirubin (mmol/L)	19.4	7.6	9.0	30.0
Total protein (g/L)	76.0	3.3	70.0	79.0
Albumin (g/L)	44.0	2.9	41.0	51.0
Globulin (g/L)	32.0	3.4	28.0	35.0
Alkaline Phosphatase (U/L)	66.3	11.6	50.0	90.0
Alanine aminotransferase (U/L)	21.8	10.5	11.0	46.0
Sodium (mmol/L)	138.1	1.1	137.0	140.0
Potassium (mmol/L)	4.6	0.3	4.2	5.1
Urea (mmol/L)	4.3	1.2	2.6	6.7
Creatinine (µmol/L)	74.5	13.7	50.0	96.0

N = 8 of which 4 males and 4 females.

Table 5. Three days average dietary intake of study participants.

**Average of three days dietary intake (per day)**				
Energy (kJ)	9996.8	3022.9	5819.9	15221.1
Energy (kcal)	2384.7	721.3	1390.75	3631.11
Protein (g)	94.3	27.6	56.5	138.2
Fat (g)	104.1	29.3	69.1	163.2
of which saturates (g)	32.2	12.0	16.4	48.7
Carbohydrates (g)	250.9	87.6	118	365.5
of which sugars (g	86.9	39.8	28.3	145.5

N = 8 of which 4 males and 4 females. Dietary intake was recorded using the *Libro, 2019* mobile application software. Intake data was analysed using the *Nutritics (2019)* Computer Software, Research Edition (v5.09).

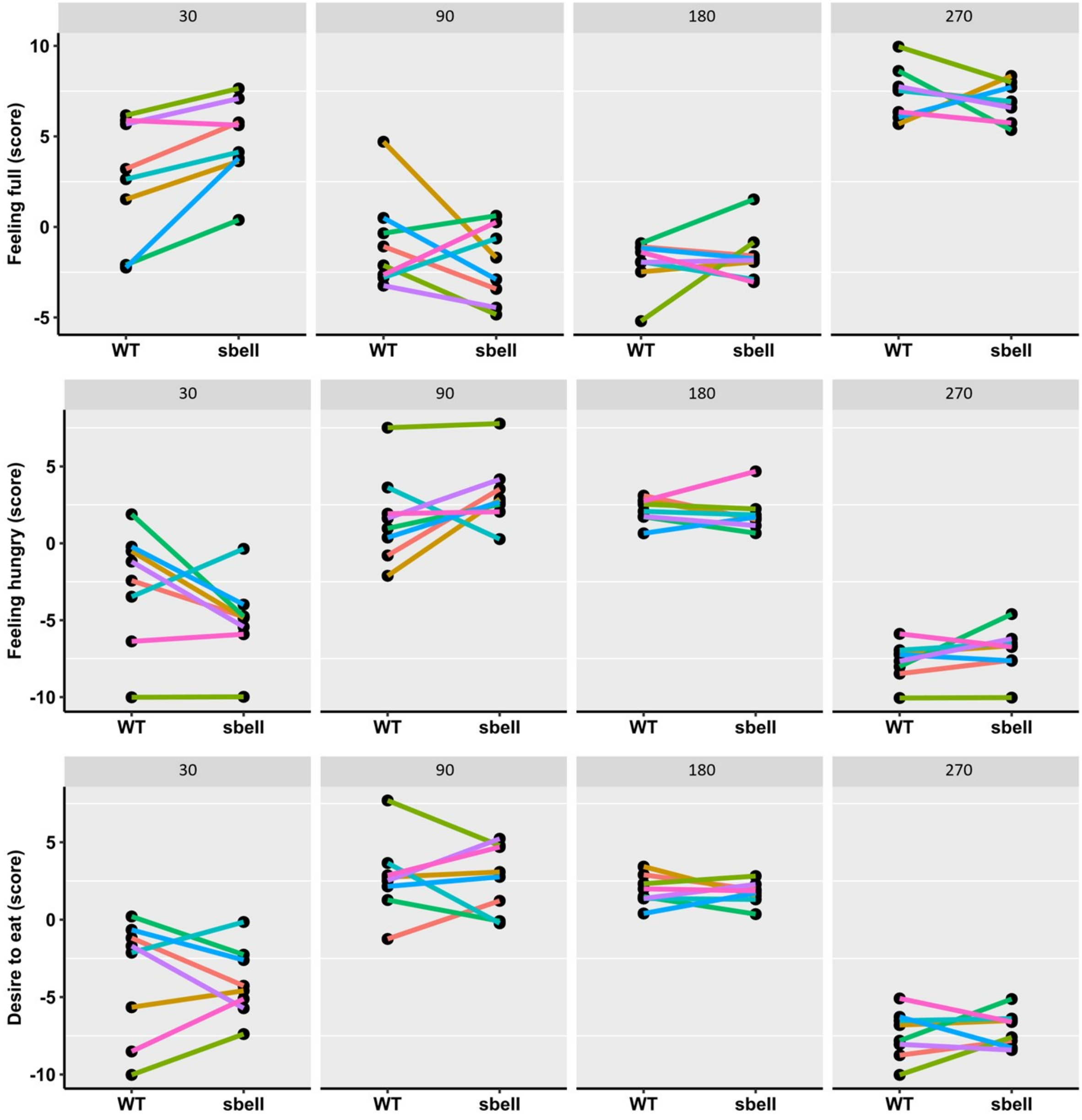

Figure 1. Individual satiety responses measured by VAS questionnaires for three main indicators reported as the change from baseline, measured at T0 (before consuming the intervention meal): 'Felling hungry', 'Feeling full', 'Desire to eat'. Change from baseline was measured at 30, 90, 180 and 270 minutes after the intervention meal.

Preliminary sensory evaluation of *sbell* bread

Sensory response to *sbell* bread were compared to the WT control bread using a hedonic scale and 'Just-About-Right' (JAR) scale. The sensory evaluation was not carried out in parallel to accommodate the requirements of the randomized crossover study design.

Method and analysis

Bread palatability was measured after each meal using a 9-point hedonic scale (1 = dislike extremely to 9 = like extremely) to determine overall likability, aroma, colour, flavour, moisture, and texture, as well as a JAR scale with five anchor points (1= much too weak to 5 = much too strong).

Two palatability questionnaires were completed after the intervention breakfast, one using a hedonic scale and one using a JAR scale. A 'penalty analysis' was used to determine which attributes of overall palatability (assessment of liking using hedonic scale) were not optimal or JAR, identifying attributes that were most detrimental to the product quality. The overall palatability scores (from the 9-point hedonic scale) were ranked against the JAR scale responses [1]. The penalty score was calculated as $Penalty\ score = \frac{not - JAR}{JAR} \times mean\ decrease\ in\ liking\ (hedonic\ score)$, where 'JAR' is the number of participants that indicated Just-About-Right and 'not-JAR' is the number of participants that indicated either too much or too little for the given attribute on the JAR scale. Any penalty scores greater than 1 indicated that improvement in the product palatability was needed.

Results and discussion

The preliminary results on palatability did not reveal major differences in bread liking. The *sbeII* bread scores for overall liking, aroma, flavour, sweetness, texture and size were above neutral on average (score = 5), as the WT control, however the *sbeII* bread appeared to be less moist than the WT control. JAR assessment suggested that participants perceived the *sbeII* bread as *drier* and *doughier* compared to the WT control but overall, it was reasonably 'acceptable' (Supplementary Figure 2).

We explored palatability of *sbeII* bread which, according to the study cohort, was reasonably acceptable compared to the WT wheat control bread roll. The sensory characteristics and consumer acceptability of *sbeII* bread has not yet been rigorously tested and requires further investigation in a non-clinical setting.

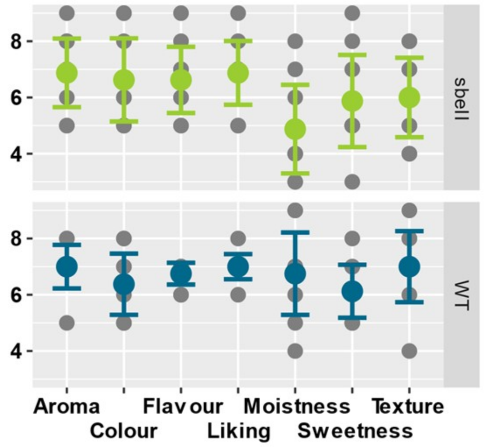

Figure 2. *Palatability scores based on sensory questionnaires, n = 8*

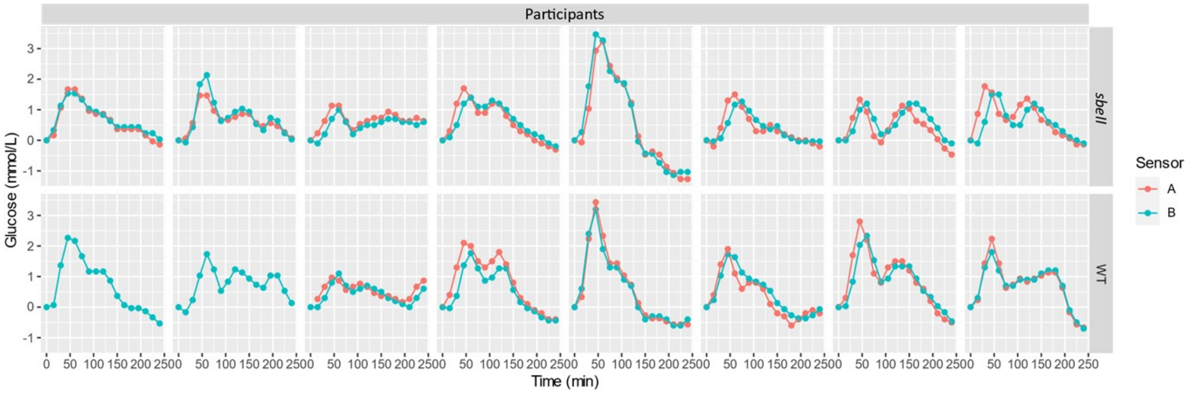

Figure 3. IF glucose response measured by CGM showing coherent response curve after subtracting the fasting glucose baseline. Two participants lost one of the two sensors and completed the study with one sensor only (bottom left of the figure).

References

1. P. Narayanan, B. Chinnasamy, L. Jin and S. Clark, Use of just-about-right scales and penalty analysis to determine appropriate concentrations of stevia sweeteners for vanilla yogurt, *Journal of Dairy Science*, 2014, **97**, 3262-3272.
